# Women empowerment and dietary diversity among Tripura Tribal women in Bangladesh

**DOI:** 10.1371/journal.pone.0339791

**Published:** 2026-01-02

**Authors:** Subarna Ghosh, Md. Mahbubur Rahman, Vhubaneshwar Tripura, Md. Zakaria Hider, Md. Shariful Islam, Sourov Ghosh Shuva, Md. Mojammel Haque Sakib, Mst. Rokshana Rabeya

**Affiliations:** 1 Department of Public Health Nutrition, Primeasia University, Banani, Dhaka, Bangladesh; 2 Department of Statistics and Data Science, Jahangirnagar University, Savar, Dhaka, Bangladesh; ICAR-CCRI: ICAR Central Citrus Research Institute, INDIA

## Abstract

**Background:**

The empowerment of women is a global concern with significant implications both for individual well-being and societal progress. This study assessed the status of women’s empowerment and its relationship with dietary diversity among Tripura ethnic women in Khagrachari district, Bangladesh.

**Methods:**

A cross-sectional survey was conducted with 230 randomly selected reproductive-aged women with a predesigned questionnaire. Significant relationships between socio-economic characteristics, women’s empowerment, women’s role in household food management, and Dietary Diversity Score (DDS) were examined by performing binary logistic regression analysis with the aid of Stata/MP 16.0 software.

**Results:**

The study findings revealed that only 13.9% (32) of the Tripura tribal women are empowered, with a mean aggregated empowerment score of 0.51 ± 0.24. Dietary assessment showed that 25% of the respondents consumed fewer than five food groups, while 15% reported no intake of Animal Source Foods (ASFs). Furthermore, several factors appear to be associated with dietary diversity and ASFs consumption of women including education, (AOR = 3.80, 95% CI: 1.66–8.69; AOR = 3.04, 95% CI: 1.23–7.48), household structure, (AOR = 4.93, 95% CI: 2.24–10.87; AOR = 3.16, 95% CI: 1.32–7.57) and meal preparation roles (ASFs; AOR = 3.30, 95% CI: 1.12–9.75). Notably, empowered women had 10.53 times higher odds (AOR = 10.53, 95% CI: 1.40–79.10) of achieving greater DDS compared to their disempowered counterparts.

**Conclusion:**

These findings highlight the importance of enhancing women’s empowerment, improving female education, and addressing household decision-making roles when designing nutrition interventions for tribal women.

## Introduction

Women’s empowerment refers to an individual’s capability to influence social and economic outcomes, exercise autonomy, and make decisions that shape their lives [1]. Despite global progress, gender inequality remains a pressing concern, highlighted by its inclusion as Goal 5 in the Sustainable Development Goals (SDGs) [2]. In Bangladesh, women’s status lags in education, health, employment, and social equality [3]. Since women constitute nearly half of the population, their empowerment is critical for the progress of the country [4]. Previous studies claimed that women’s empowerment improves household and child nutrition [5–7]; however, little is known about whether empowerment influences women’s own dietary diversity [4]. This gap is significant given that about 54% of Bangladeshi women of childbearing age consume fewer than four food groups daily, reflecting poor dietary diversity [8].

Inadequate dietary intake, along with lower Animal Source Foods (ASFs) consumption, may cause severe protein-energy malnutrition and related micronutrient deficiency disorders [9,10]. While empowerment has been linked to household-level food access [11,12], its role in shaping individual women’s dietary practices remains unexplored. This knowledge gap is particularly relevant for Bangladesh’s tribal and indigenous populations. The country hosts nearly 150 tribal groups [13], where about 44.4% of the ethnic population, with eleven ethnic multi-lingual minorities concentrated in the Chittagong Hill Tracts (CHTs). One such indigenous community is the Tripura tribe, also recognized as Tripuri, Tiprah, or Tipperah. The Tripura tribe is predominantly Hindu and resides mainly in the Chittagong and Sylhet divisions of the country.

Their livelihood depends primarily on jhum cultivation, which is usually subsistence-oriented, producing foods for household consumption, though surplus crops occasionally are sold in local markets [14]. However, many research articles reported that the ethnic communities of CHTs remain underprivileged due to limited access to income, education, health services, appropriate housing conditions, and pure water sources [15–17]. A comparatively higher proportion of CHT households borrow money to purchase food compared to rural Bangladeshi households, reflecting their difficulty in accessing nutrient-enriched foods [18]. Moreover, collecting information from these areas is challenging because of difficult terrain, linguistic barriers, geographical isolation, and insecurity.

Consequently, the situation of women in tribal communities remains inadequately addressed. Although Bangladesh’s growing economy may influence women’s status and the dietary habits of ethnic minority groups, the determinants of women’s empowerment and dietary diversity in tribal areas remain underexplored. Therefore, this study investigates impact of women’s empowerment on dietary diversity within the Tripura tribal community of Bangladesh. Specifically, it assesses the extent of empowerment among Tripura women and examines how empowerment shapes their food habits. By revealing how empowerment influences diet, this study seeks to support strategies that strengthen women’s nutrition and well-being in marginalized tribal communities.

## Materials and methods

This cross-sectional study was conducted on the Tripura minority ethnic group in Khagrachari Hill district, Chittagong division, Bangladesh. Data were collected from September to December 2022 from three conveniently selected wards: Bhaibonchhara (ward no. five) and Perachhara (wards no. seven and eight) union of Khagrachari district. Given the geographic isolation and logistical challenges of working in the Chittagong Hill Tracts, convenience selection of study areas was the most feasible approach to ensure an adequate sample size and reliable data collection within the study’s time and resource constraints. The study population consisted of married women of reproductive age from the Tripura community. According to the Population and Housing Census 2022, an estimated 176721 tribal women live in the Khagrachari district. [19]. Besides this, the 5DE for Bangladesh shows that 39 percent of women are empowered [20]. Based on these parameters, the study applied Cochran’s formula to calculate the sample size for the finite population.


$$\text{Sample}\;\text{size},\;\text{n}=\frac{\;n_{\text{0}}}{\text{1}+\;\frac{\left(n_{\text{0}}-\text{1}\right)}{N}}$$

Here, n_o_ = z^2^pq/d^2^. At a 95% confidence interval, z = 1.96, expected prevalence, p = 39%, q= (1-p) =0.61, and marginal error, d = 7%. So, n_o_= (1.96^2^ * 0.39*0.61)/ (0.07)^2^ = 187. The sample size for this study is


$$\text{n}=\frac{\;\text{1}\text{8}\text{7}}{\text{1}+\;\frac{\left(\text{1}\text{8}\text{7}-\text{1}\right)}{\text{176721}}}=\text{186}.\text{81}=\text{187}$$

The estimated sample size is 187. To account for an anticipated 19% non-response rate due to invalid or incomplete responses, an additional 43 samples were added. Finally, data were collected from 230 reproductive-aged tribal women.

According to the WHO, women of reproductive age refer to all women aged 15–49 years [21]. First of all, a list of married reproductive-aged women was prepared with the help of the Union Parishad chairman and the head of the village. Thereafter, a simple random sampling technique was used to select 230 women from them. A written, structured questionnaire, based on the study objectives, was prepared for data collection, where both closed-ended and open-ended questions were applied. The entire interview was conducted in the native language (Kokborok language) with the help of interpreters. Additionally, written consent was taken from the respondents before data collection. This study also received approval from the Institutional Ethical Approval Committee (IEAC) of Primeasia University, Bangladesh. The approval number is PAU/IEAC/22/105.

Inclusivity in global research: Additional information regarding the ethical, cultural, and scientific considerations specific to inclusivity in global research is included in the Supporting Information (S1 Checklist).

The study investigated socioeconomic and demographic conditions, Dietary Diversity Score (DDS), Animal Source Foods (ASFs) consumption, and women’s empowerment status in the study area. The Women’s Empowerment Agricultural Index (WEAI) tool was used to measure women’s empowerment in the Tripura tribal community [20]. The five domains of WEAI, represented in S1 Table, include (i) decisions about agricultural production, (ii) access to and decision-making power about productive resources, (iii) Control of use of income, (iv) Leadership in the community, and (v) Time allocation. The adequacy and inadequacy score of indicators of each domain was measured by weighting as per the guidelines of Alkire et al [20]. Then the inadequacy score is calculated by summing the weighted inadequacies experienced by each person. The formula for calculating the individual indicator score- ci* *= *w*1*I*1 + *w*2*I*2 + ... + *wdId*, where *Ii* = 1 if the person has an inadequate achievement in indicator *i* and *Ii* = 0 otherwise, and *wi* is the weight attached to indicator *i* with $\sum_{i=\text{1}}^{d}\text{wi}=\text{1}$. The score lies between 0 and 1 and an individual is disempowered if her inadequacy score (c_i_) is greater than >0.2 and empowered if the score is ≤ 0.2.

Dietary Diversity Scoring (DDS) is a qualitative measure of the consumption of food that reflects nutrient adequacy and access to diverse foods for individuals. Dietary diversity was assessed using dietary recall, in which respondents were requested to recall their exact food intake over the previous 24 hours. A predesigned questionnaire for Women’s Dietary Diversity Score (WDDS) established by the Food and Agriculture Organization (FAO) was used for this study [22]. The interview schedule included nine food groups as per the cited dietary diversity assessment guidelines.

When asked about their food intake over the previous 24 hours, the women answered whether or not they had eaten anything from the listed food groups. There were zero to nine distinct food groups included in the overall number of meals. Scores below five were considered inadequate dietary diversity, while scores of five or above indicated adequate dietary diversity. The consumption of ASFs was also measured by observing the meat, eggs, or fish consumption of women in the previous 24 hours. Thereafter, food consumption from animal sources was considered adequate, while no food intake from animal sources was denoted as inadequate ASF consumption.

For the analysis, data were inputted into IBM SPSS Statistics 22 software and then transferred to Stata/MP 16.0 software. The distribution of dietary diversity scores of women according to their socio-economic status and role in household food management was observed by using a chi-square (χ2) test. The adjusted binary logistic regression (Enter model method) analysis was used to identify the factors associated with the dietary diversity status and animal food consumption of tribal women. Multicollinearity and model fitness were checked by the Variance Inflation Factor (VIF) and the Hosmer and Lemeshow goodness of fit test. The strengths of the associations were observed by estimating the Odds Ratios (ORs) at a 95% Confidence Interval (CI), and statistical significance was assessed at a p-value of less than 0.05. Finally, the association between dietary diversity score and women’s empowerment was demonstrated by Pearson’s correlation coefficient and scatter plot.

## Results

The socio-economic status of Tripura tribal women (n = 230) indicates that nearly half of the respondents, 49.1% (113), were aged between 26 and 35 years, followed by 44.3% (102) in the 18–25 years age group. The mean age was 27.21 ± 5.32. Regarding educational qualifications, a significant proportion of the participants, 47.4% (109), had primary education, whereas 24.8% (57) of women had no formal education. In the Tripura tribal community, most participants, 57% (131), belonged to households that comprised four or fewer members. The majority of the women, 67.8% (156) of that area, were involved with agriculture, although 60% (138) reported a monthly income below 5000 BDT.

The study also looked at how tribal women manage household food allocation and found that 55.7% (128) contributed financially to the purchase of family food. A considerable percentage of tribal women were responsible for meal preparation (90.9%) and for the distribution of meals (95.7%) among family members. Bivariate analysis showed that adequate dietary diversity was more common among women aged 26–35 years (χ^2^ = 8.72, *p* = 0.013), those with primary education (χ2 = 16.21, *p* = 0.001), those involved in agricultural activities (χ2 = 7.47, *p* = 0.024), and those living in households with four or fewer members (χ2 = 15.97, *p* < 0.001). Additionally, women who contributed financially to food purchasing (χ2 = 14.08, *p* < 0.001) and who were involved in food preparation (χ2 = 9.04, *p* = 0.006) had higher dietary diversity (Table 1).

**Table 1:** Distribution of dietary diversity scores of women according to their socio-economic status and role in household food management (n = 230).

Characteristics	Total	Dietary Diversity		Chi-square test	
	n (%)	Inadequate n (%)	Adequate n (%)	χ2 (df)	p-value
**Age group**					
18–25 years	102 (44.3%)	35 (60.3%)	67(39.0%)	8.72 (2)	0.013
26 - 35 years	113 (49.1%)	19 (32.8%)	94 (54.7%)		
≥ 36 years	15 (6.5%)	4 (6.9%)	11 (6.4%)		
**Age (Mean ± SD)**	27.21 ± 5.32	26.38 ± 5.893	27.49 ± 5.098	**–**	**–**
**Education**					
No institutional education	57 (24.8%)	25 (43.1%)	32 (18.6%)	16.21 (3)	0.001
Primary education	109 (47.4%)	24(41.4%)	85 (49.4%)		
Secondary education	31 (13.5%)	6(10.3%)	25 (14.5%)		
Higher education	33 (14.3%)	3(5.2%)	30 (17.4%)		
**Occupation**					
Housewife	29 (12.6%)	11 (19%)	18 (10.5%)	7.47	0.024
Agriculture	156 (67.8%)	42 (72.4%)	114 (66.3%)		
Job holder	45 (19.6%)	5 (8.6%)	40 (23.3%)		
**Income**					
No income	29 (12.6%)	11 (19%)	18 (10.5%)	4.55 (2)	0.103
Below 5k	138 (60%)	36 (62.1%)	102 (59.3%)		
≥ 5k	63 (27.4%)	11 (19%)	52 (30.2%)		
**Family size**					
≤ 4 members	131 (57%)	20 (34.5%)	111 (64.5%)	15.97 (1)	<0.001
> 4 members	99 (43%)	38 (65.5%)	61 (35.5%)		
**Financial contribution to food purchasing**					
No	102 (44.3%)	38 (65.5%)	64 (37.2%)	14.08 (1)	<0.001
Yes	128 (55.7%)	20 (34.5%)	108 (62.8%)		
**Food Preparation**					
No	21 (9.1%)	11 (19%)	10 (5.8%)	9.04 (1)	0.006
Yes	209 (90.9%)	47 (81%)	162 (94.2%)		
**Food distribution**					
No	10 (4.3%)	2 (3.4%)	8 (4.7%)	.15 (1)	1.000
Yes	220 (95.7%)	56 (96.6%)	164 (95.3%)		

Table 2 represents the adequacy achievement of tribal women across five domains of empowerment. The findings show that 43.5% (100) of women reported being able to make decisions about agricultural production, while 57% (131) lacked autonomy to grow crops independently. Although 62.6% (144) of tribal women owned property, nearly half, 50.4% (116) of them don’t have the authority to purchase or sell any assets. By contrast, a majority, 77.4% (178), had control over their earnings. In the study area, only 13% (30) of women are involved with social groups or community organizations, yet 27.4% (63) of women are unable to raise their voice in public gatherings. Besides this, 63.5% (146) of women reported dissatisfaction with their leisure time due to long working hours. The aggregated empowerment score further highlights these challenges. The mean score was 0.51 ± 0.24, with only 13.9% (32) of women classified as empowered.

**Table 2:** Status of women’s empowerment in the Tripura community.

Domains	Indicators	Adequacy		Inadequacy	
		Frequency	Percentage	Frequency	Percentage
Production	Input in productive decisions	100	43.5	130	56.5
	Autonomy in production	99	43	131	57
Resources	Ownership of assets	144	62.6	86	37.4
	Purchase, sale, or transfer of assets	114	49.6	116	50.4
	Access to and decisions about credit	30	13	200	87
Income	Control over the use of income	178	77.4	52	22.6
Leadership	Group member	30	13	200	87
	Speaking in public	167	72.6	63	27.4
Time	Workload	89	38.7	141	61.3
	Leisure	84	36.5	146	63.5
Women empowerment status		**Disempowered**		**Empowered**	
		**Frequency**	**Percentage**	**Frequency**	**Percentage**
		198	86.1	32	13.9
Aggregate empowerment (5DE) [1 = disempowered]		0.51 ± 0.24			

**Fig 1:**
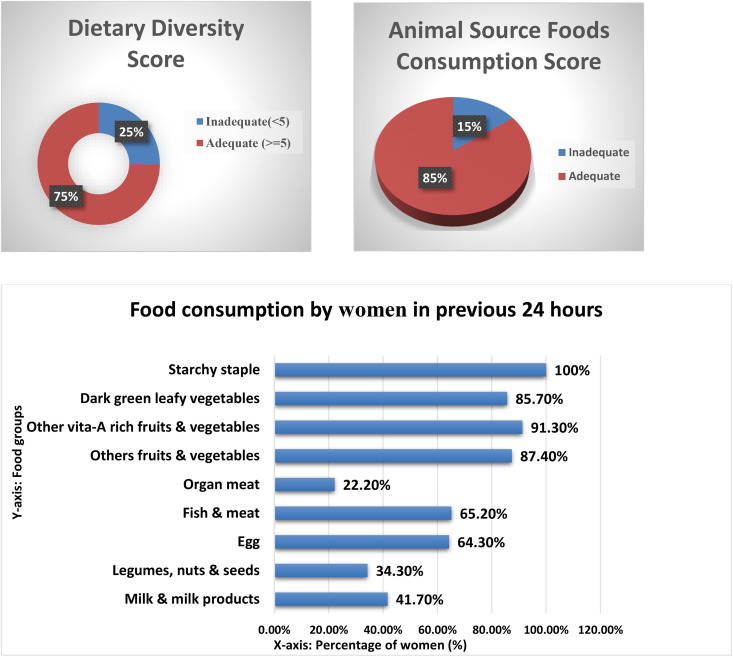
Dietary diversity status of tribal women.

Regarding the DDS of tribal women, 25% (58) of respondents consume food from fewer than five food groups, while the mean DDS was 5.9 ± 1.7. In terms of ASFs, 85% (195) of women reported consuming at least one animal source food in the previous 24 hours. Findings from the 24-hour dietary recall indicate that all of the women in this sample consumed starchy foods (grains, tubers, and roots). Fruits and vegetables rich in vitamin A were the second most commonly consumed group, reported by 91.3% of participants. Furthermore, a considerable percentage of women consumed leafy vegetables (85.7%), eggs (64.3%), and, fish and meat (65.2%). In contrast, organ meats (22.2%) and milk products (41.7%) were the least frequently consumed food groups (Fig 1).

A binary logistic regression analysis was carried out to look at the factors influencing the dietary diversity status of tribal women (Table 3). The Hosmer and Lemeshow test statistics indicated a good model fit both for DDS (χ2 = 9.545; df = 8; *p* = 0.635) and ASFs consumption (χ2 = 15.136; df = 8; *p* = 0.124). The adjusted model of regression analysis demonstrates that the DDS score of women was significantly influenced by their age and educational status, family size, role in household food management, and women’s empowerment. The study findings indicate that the women aged 26–35 years had higher odds of achieving adequate DDS compared with women under 25 years (AOR = 4.10, 95% CI: 1.78–9.39). On the contrary, tribal women older than 35 years had 92% lower odds of consuming ASFs (AOR = 0.08, 95% CI: 0.01–0.58). Education also played a strong role in women’s DDS. Women who completed primary education were 3.8 times more likely to achieve higher DDS (AOR = 3.80, 95% CI: 1.66–8.69) and 3.04 times more likely to consume ASFs (AOR = 3.04, 95% CI: 1.23–7.48) compared to the women who did not have any institutional education.

**Table 3:** Regression analysis of factors influencing the dietary diversity of Tribal women.

Indicators	Dietary Diversity Score			Animal Source Foods		
	AOR	p-value	95% CI	AOR	p-value	95% CI
**Age group**						
18–25 years	Reference			Reference		
26 - 35 years	4.10	**0.001**	1.78-9.39	1.71	0.244	0.69 −4.25
≥ 36 years	0.321	0.185	0.06-1.71	0.08	**0.012**	0.01-0.58
**Education**						
No institutional education	Reference			Reference		
Primary education	3.80	**0.002**	1.66- 8.69	3.04	**0.015**	1.23-7.48
Secondary education	2.97	0.090	0.84- 10.46	4.11	0.101	0.75- 22.28
Higher education	3.65	0.113	0.73-18.07	2.32	0.347	0.40-13.53
**Occupation**						
Housewife	Reference			Reference		
Agriculture	1.30	0.819	0.13- 12.65	1.94	0.643	0.11- 32.76
Job holder	2.58	0.477	0.18 - 35.37	3.66	0.426	0.15- 89.18
**Income**						
No income	Reference			Reference		
Below 5k	0.61	0.675	0.06- 6.06	0.52	0.653	0.03- 8.83
≥ 5k	0.40	0.480	0.03-4.91	0.37	0.524	0.01 - 7.73
**Family size**						
> 4 members	Reference			Reference		
≤ 4 members	4.93	**<0.001**	2.24-10.87	3.16	**0.010**	1.32-7.57
**Financial contribution to food purchasing**						
No	Reference			Reference		
Yes	3.36	**0.005**	1.43- 7.88	2.51	0.064	0.94-6.66
**Food Preparation**						
No	Reference			Reference		
Yes	2.78	0.065	0.93- 8.25	3.30	**0.030**	1.12- 9.75
**Food distribution**						
Yes	0.55	0.534	0.08-3.60	0.49	0.564	0.04-5.40
**Women empowerment**						
No	Reference			Reference		
Yes	10.53	**0.022**	1.40 −79.10	20.08	**0.033**	1.27 - 316.64

NB: AOR = Adjusted Odds Ratio, CI = Confidence Interval, Variance Inflation Factor (VIF) = 1.53, Significance level 5%.

Household structure was another significant indicator. Women from families with fewer than five members have higher odds for DDS (AOR = 4.93, 95% CI: 2.24–10.87) and ASFs consumption (AOR = 3.16, 95% CI: 1.32–7.57) compared to women who belong to an extended family. The DDS score is also higher among the women who purchase food (AOR = 3.36, 95% CI: 1.43–7.88) for the family. Additionally, ASFs consumption (AOR = 3.30, 95% CI: 1.12–9.75) are greater among the women who prepare meals for family members. Finally, empowerment emerged as a critical determinant for DDS. Empowered tribal women were 10.53 times more likely to have higher DDS (AOR = 10.53, 95% CI: 1.40–79.10) and 20.08 times more likely to consume ASFs (AOR = 20.08, 95% CI: 1.27–316.64) compared to the women who were not empowered in the study area.

**Fig 2:**
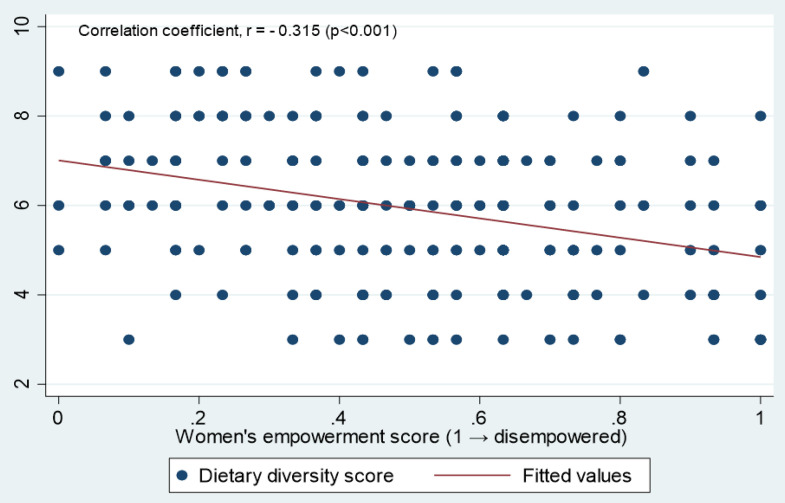
The correlation between dietary diversity score and women’s empowerment.

In Fig 2, the correlation coefficient (r = −0.315, p < 0.001) demonstrates a statistically significant inverse correlation between women’s empowerment score and DDS. Since the empowerment score ranges from 0 to 1, with values greater than >0.2 denoting disempowerment, the negative coefficient suggests higher levels of disempowerment are significantly associated with lower dietary diversity.

## Discussion

The study was conducted among reproductive-aged women of the Tripura tribe of Bangladesh to assess their level of empowerment and dietary diversity patterns. The study revealed that the majority of tribal women have either no formal education or only primary level schooling, which confines them to subsistence farming or low-income activities, typically earning less than 5000 BDT per month. Agriculture is the mainstay of tribal women’s livelihood, with 67.8% of women in that area solely engaged in agriculture. However, their decision-making about farming remains limited; only 43.5% report adequacy in production decisions, and 43% have autonomy in crop selection. The study findings align with the Bangladesh Demographic and Health Survey-2014 report, which notes that 44% of Bangladeshi women can make decisions on household expenditure, healthcare, and visiting relatives, while only 8% influence major household purchases [23]. It implies that the factors shaping solidarity and decision-making power among tribal women appear comparable to those affecting rural Bangladeshi women.

This study also assessed women’s empowerment across five domains and found an average score of 0.51 ± 0.24 on a 0–1 scale. For comparison, Alkire et al. [20] reported a weighted average of the 5DE sub-index value of 0.746 for women in Bangladesh, while the Tripura tribal community in India scored 0.83 [24]. According to this study, 13.9% of women of the Tripura tribe of Bangladesh are empowered, which is notably lower than Bangladesh overall (39%) and the Indian Tripura tribal (70%) community [20,24]. Globally, women’s empowerment level varies widely; for instance, 25% of women are empowered in Mali, compared with 96.7% in Zimbabwe [25]. It indicates that empowerment among the Tripura tribal community in Bangladesh is much lower than in other contexts. The authors attribute this gap to hard-to-overcome factors such as geographical isolation, slow economic progress, and limited access to essential services, which may restrict tribal women’s ability to achieve self-reliance and empowerment.

The analysis of DDS data revealed that most tribal women have high dietary diversity with a mean WDDS score of 5.9 ± 1.7. On the other hand, a study assessed DDS of tribal women in northern Bangladesh and reported a much lower mean DDS of 3.25 ± 0.81 [26]. Similarly, studies conducted among rural women in Bangladesh found mean DDS values ranging from 3.8 to 4.14 [27,28]. The dietary pattern of tribal women is largely shaped by available natural food sources, many of which are directly collected from forests [29]. The higher DDS of tribal women reflects the availability of indigenous vegetables and animals, which provide a wide variety of food options. The abundance of local food enables these women to consume foods from more than four food groups, including intake of starchy staples, vitamin-rich fruit and vegetables, and animal food sources in a higher proportion.

After assessing dietary patterns, the study focused on influencing factors of dietary diversity of tribal women and revealed that middle-aged women have higher DDS, while older women consume fewer animal-source foods. Consistent with previous studies [30,31], this study also observed higher dietary diversity and animal-source food consumption among the women who have institutional education. This study’s findings also align with the study of Kundu et al. [32], where it is claimed that the nuclear family had greater DDS compared to the extended families. Furthermore, several studies [27,33,34] highlighted that women’s decision-making capacity, access to the market, and control over the food budget improve dietary diversity by reducing household food insecurity. Likewise, it is also evident in our study that women who contribute to family food purchasing have a higher DDS. Additionally, women who prepare family meals were more likely to consume diverse foods, including animal-source products. Nevertheless, different studies have found positive associations between diversity in household diet and women’s aggregate empowerment in Bangladesh [1,4,35–38]. Our findings reinforce this evidence, showing that women’s empowerment is significantly and positively associated with dietary diversity and animal-source food consumption of tribal women, even after adjusting for other influencing factors.

### Strengths and limitations of the study

The major strength of the research is the random selection of participants from an ethnically homogenous population, providing robust evidence on women’s empowerment and nutrition outcomes in tribal Bangladesh, a context that has been largely overlooked in previous research. The limitations include the inability to assess gender gaps using the Gender Parity Index (GPI) sub-index and domain-specific associations with DDS. Furthermore, inaccessible terrain and language barriers restricted the measurement of household food security and the quantity of food consumption. A broader dietary assessment was needed to strengthen the understanding of the empowerment-nutrition relationship in tribal communities. Future research should incorporate GPI to capture gender disparity more comprehensively and apply domain-specific analyses to clarify which aspects of women’s empowerment drive improvement in women’s DDS.

## Conclusion

The study highlights the critical link between women’s empowerment and DDS, emphasizing that disempowered tribal women tend to have poor DDS. It suggests that policymakers could use WEAI domains to identify the most critical areas of disempowerment, such as decision-making, access to resources, or income control, and target interventions accordingly. Strengthening women’s agency in ethnic areas may not only advance gender equity but also improve their nutritional status. Additionally, the study underscores the importance of education and training in fostering women’s empowerment. Therefore, ensuring education and skill-building opportunities for women in ethnic communities, particularly in areas like food purchasing and meal preparation, will improve their DDS and overall health outcomes.

## Supporting information

S1 Table Measurement of women’s empowerment score. (DOCX)

S1 Checklist Inclusivity-in-global-research-questionnaire. (DOCX)
