# Supplementary material for: Women empowerment and dietary diversity among Tripura Tribal women in Bangladesh
Source: PLoS One. 2026 Jan 2;21(1):e0339791. doi: 10.1371/journal.pone.0339791 (PMC12758706; doi:10.1371/journal.pone.0339791)
Supplement: S1 Table — (DOCX) [file pone.0339791.s001.docx]

**S1 Table:** Measurement of Women's Empowerment Score

| **Domain** | **Indicator** | **Adequacy cut-off** | **Weight** |
| --- | --- | --- | --- |
| Production | Input in productive decisions | If she participates and has at least some input in agricultural decisions | 1/10 |
|  | Autonomy in  Production | If her actions are relatively more motivated by her own values | 1/10 |
| Resources | Ownership of  Assets | If she reports having sole or joint ownership of at least one major asset | 1/15 |
|  | Purchase, sale, or  transfer of assets | If she participates (or can participate) in decisions to buy, sell, or transfer the asset, conditional on the household owning it. | 1/15 |
|  | Access to and  decisions about credit | If she has access to credit, or she can participate in at least one decision regarding credit access | 1/15 |
| Income | Control over use  of income | If she has input into decisions about income generated, conditional on participation in the activity. | 1/5 |
| Leadership | Group member | If she is a member of at least one group | 1/10 |
|  | Speaking in public | If she is comfortable speaking up in public or responding to questions related to infrastructural development. Wages, misbehavior of authorities, or other concerning issues. | 1/10 |
| Time | Workload | If she worked less than 10.5 hours in the previous 24 hours. | 1/10 |
|  | Leisure | Satisfied with the time available for leisure | 1/10 |
